# The Effects of Maxillary Protraction with or without Rapid Maxillary Expansion and Age Factors in Treating Class III Malocclusion: A Meta-Analysis

**DOI:** 10.1371/journal.pone.0130096

**Published:** 2015-06-11

**Authors:** Wei Zhang, Hong-Chen Qu, Mo Yu, Yang Zhang

**Affiliations:** 1 Department of Orthodontics, School of Stomatology, China Medical University, Shenyang, Liaoning, P.R. China; 2 Department of Urological Surgery, The Fourth Affiliated Hospital of China Medical University, Shenyang, Liaoning, P.R. China; New York University, UNITED STATES

## Abstract

We conducted a comprehensive meta-analysis of 12 studies to examine whether maxillary protraction face mask associated with rapid maxillary expansion (FM/RME) could be an effective treatment for Class III malocclusion and to evaluate the effect of timing on treatment response. Patients with a maxillary deficiency who were treated with FM with or without RME were compared with those who had an untreated Class III malocclusion. In both treatment groups, forward displacement of the maxilla and skeletal changes were found to be statistically significant. In addition, posterior rotation of the mandible and increased facial height were more evident in the FM group compared with the control group. However, no significant differences were observed between the early treatment groups and late treatment groups. The results indicated that both FM/RME and FM therapy produced favorable skeletal changes for correcting anterior crossbite, and the curative time was not affected by the presence of deciduous teeth, early mixed dentition or late mixed dentition in the patient.

## Introduction

Mandibular or mandibular dentition prognathism, retrusive maxillary or maxillary dentition, and combinations of these components may lead to a Class III malocclusion[1–5]. According to surveys, seventy-five percent of skeletal Class III malocclusions are caused by maxillary retrognathism or a combination of maxillary retrognathism and mandibular prognathism. Several authors have agreed that maxillary retrusion is the most common contributing component of Class III features[5,6]. Because the possibility of Class III malocclusion characterized by maxillary hypoplasia should be considered, it has become more important to use devices that encourage maxillary growth. Several techniques have been described to effectively protract the maxilla, including the use of a face mask (FM) or reverse chin cup and the application of direct force via ankylosed primary canines[7–13]. In addition, miniplate and miniscrew implants have also been used to provide the necessary orthodontic anchorage in patients with retrusive maxillary dentition[14–19]. The treatment of a skeletal Class III malocclusion is challenging for orthodontics, primarily because of the concave profile of the midface and the unpredictable growth potential of the maxilla coupled with potentially unfavorable mandibular growth.

Current non-surgical treatment methods for severe skeletal Class III malocclusions to correct maxillary discrepancies in young adolescents include rapid maxillary expansion (RME)[20–23]. However, maxillary advancement through the application of extra-oral orthopedic force is considered a viable treatment option in developing children. As a result, FM protraction therapy also has been advocated during the early treatment of Class III malocclusion with maxillary deficiency. A FM is a device commonly used to interfere with growing class III malocclusions with maxillary deficiency, and the use of FM to encourage maxilla growth has gained popularity among orthodontists over the last 30 years. However, the real skeletal encouragement of maxilla growth over time from this traditional method has been debated and remains controversial. Most skeletal Class III malocclusions include disharmony in terms of the length and width of the maxilla, which can be corrected by a rapid maxilla expander. Therefore, we want to provide the best evidence and further persuasive data to confirm the validity of FM and to determine whether the combination of FM/RME is an effective method to improve anterior crossbite. This systematic literature review was also conducted to determine whether an early treatment time is the optimal period to begin FM treatment because an earlier treatment start might lead to more growth compared with that of late controls.

## Materials and Methods

### 1 Literature search

We used [‘face mask’ or ‘FM’], [‘face mask/rapid maxillary expansion’ or ‘FM/RME’ (text word)], [Class III malocclusion or Angle Class III (MeSH)] and [‘maxillary protractor’] as search terms. The wide electronic search scope included PubMed, Cochrane Library, Web of Science, Springer Link, and ScienceDirect. In addition, we searched all these databases to avoid missing relevant studies published before October 6, 2014. We also evaluated studies that were cited in the reference lists of the included papers to ensure the inclusion of all relevant studies.

### 2 Inclusion and exclusion criteria

The publications had to reach the following standards to meet the strict inclusive criteria: i) the study concentrated on the treatment efficacy of FM or FM/RME and the relationship between timing factors and maxillary protraction; ii) all patients had clinical Class III malocclusion from the period of early mixed dentition to early permanent dentition, and their ages ranged from seven to fourteen years old; iii) the study provided the original data, or we were able to obtain the data from the primary data; and iv) the study was a case-control study or a randomized controlled trial (RCT). Moreover, the language of all included studies had to be English. We required complete, accurate, and useful data; consequently, reviews, abstracts, conference papers, case reports and letters were excluded without consideration.

### 3 Data extraction

We extracted information from the included research, such as author names, publication year, volume and issue; article design; number of cases and placebos, efficacy and safety assessment. Wei Zhang and Hong-Chen Qu independently checked the data from all the included studies. Subsequently, a third reviewer (Yang Zhang) discussed inconsistent evaluations and thereby helped to reach a final agreement.

### 4 Quality assessment of the included studies

Each publication’s quality was assessed by two reviewers (Wei Zhang and Hong-Chen Qu) according to a modified STROBE quality score system [24, 25]. The quality-assessment scores ranged from 0 to 44. Scores of 0–17.5 were regarded as poor, 7.5–35 as fair, and 35–44 as good. Subsequently, the two reviewers met to discuss disagreements and draw a final reasonable conclusion.

### 5 Statistical analysis

To acquire reliable and accurate results, two authors (Mo Yu and Yang Zhang) who were not involved in the data collection were in charge of extracting the data. The authors calculated the mean difference (MD) and 95% CI using Review Manager Version 5.3 software (provided by the Cochrane Collaboration). The I^2^ test was used to quantify the effect of heterogeneity. A higher result on the I^2^ test represented an increased possibility that heterogeneity contributed to the inter-study variability. Both fixed-effects and random-effects models were used: if the I^2^ test < 50% or P ≥ 0.05 (Q-test), we used the fixed-effects model; if there was significant heterogeneity among the included studies (I^2^ test > 50%), the random-effects model was employed. We used Funnel plots to detect publication bias; a symmetrical plot indicated little publication bias.

## Results

### 1 Characteristics of the included studies

**Fig 1:**
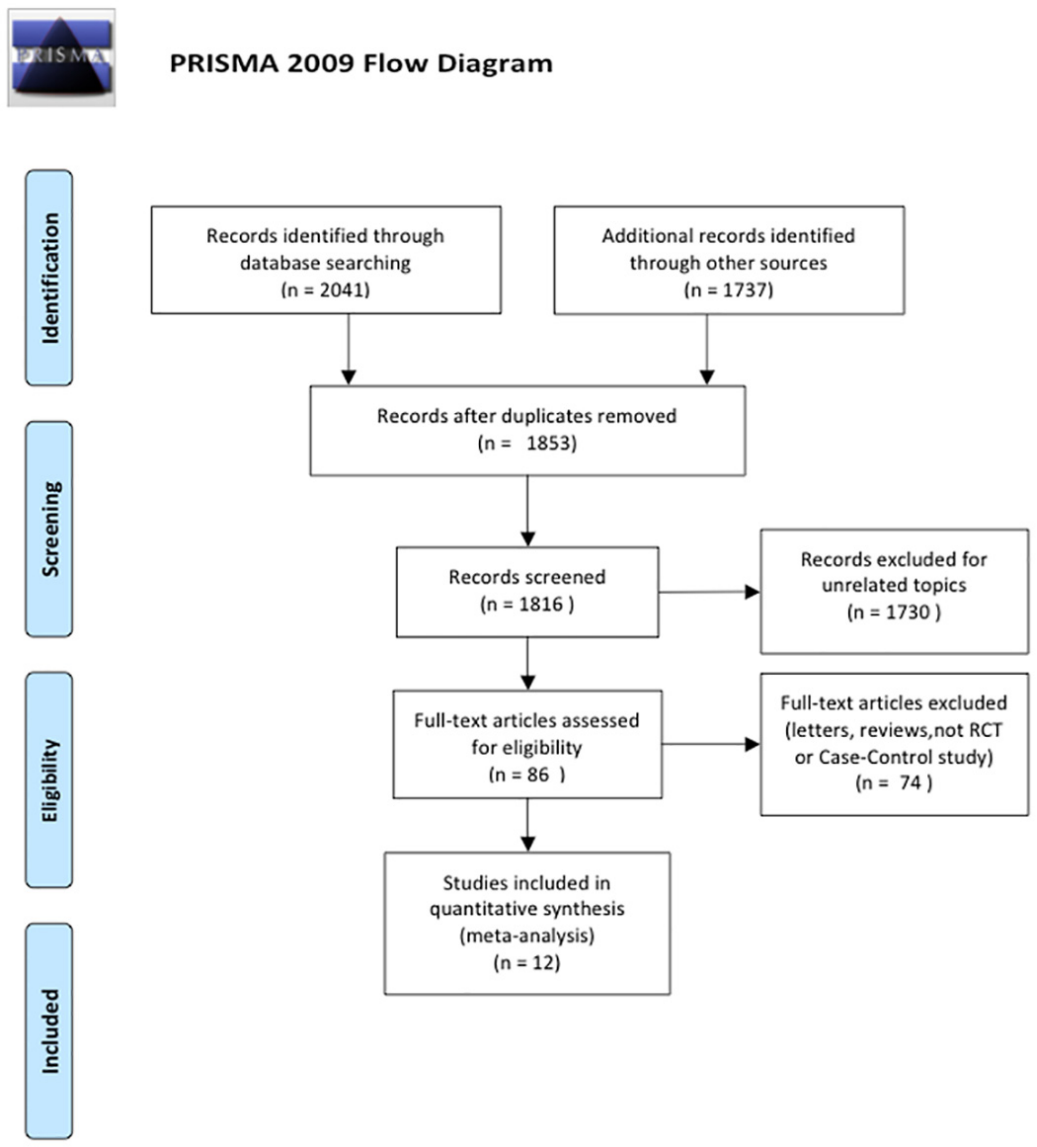
The flow chart of study selection. In this meta-analysis, 12 studies were selected for qualitative analysis.

Based on the inclusion criteria, we included 12 satisfactory studies[26–37] in the meta-analysis. Fig 1 shows the flow chart of study selection. The 12 studies included 318 patients with retruded maxilla characterized by Class III malocclusion and 228 untreated Class III malocclusion control patients. The studies included were published from 1998 to 2014. All treatment groups received the maxilla protractor with or without rapid maxillary expansion. The control patients were defined as having Class III malocclusion using cephalometric angular and linear parameter analysis. The baseline quality score of the involved studies was 17.5 (fair or good). Table 1 illustrates the methodological quality and characteristics of the included studies.

**Table 1:** Characteristics and methodological quality of the included studies.

Author	Year	Number	Study design	Quality score
Gencer	2014	FM group(n = 15); Control(n = 15)	Case-Control	28
Chen	2012	FM group(n = 22); Control(n = 17)	Case-Control	24
Ucem	2004	FM group(n = 14); Control(n = 14)	Case-Control	25
Kilicoglu	1998	FM/RME group(n = 16); Control (n = 10)	Case-Control	23
Masucci	2011	FM/RME group(n = 22); Control (n = 16)	Case-Control	26
Sar	2011	FM/RME group(n = 15); Control (n = 15)	Case-Control	24
Yuksel	2001	FM/RME group(n = 34); Control (n = 17)	Case-Control	26
Kajiyama	2000	FM/RME group(n = 29); Control (n = 25)	Case-Control	22
Lee	2010	Early treatment group (n = 26); Late treatment group (n = 23)	Case-Control	27
Franchi	2004	Early treatment group (n = 33); Late treatment group (n = 14)	Case-Control	24
Kajiyama	2004	Early treatment group (n = 63); Late treatment group (n = 57)	Case-Control	24
Baccetti	2000	Early treatment group (n = 32); Late treatment group (n = 28)	Case-Control	25

FM: face mask therapy. FM/RME: face mask and rapid maxillary expansion therapy

### 2 Differences in FM-treated Class III malocclusion patients group and untreated controls

**Fig 2:**
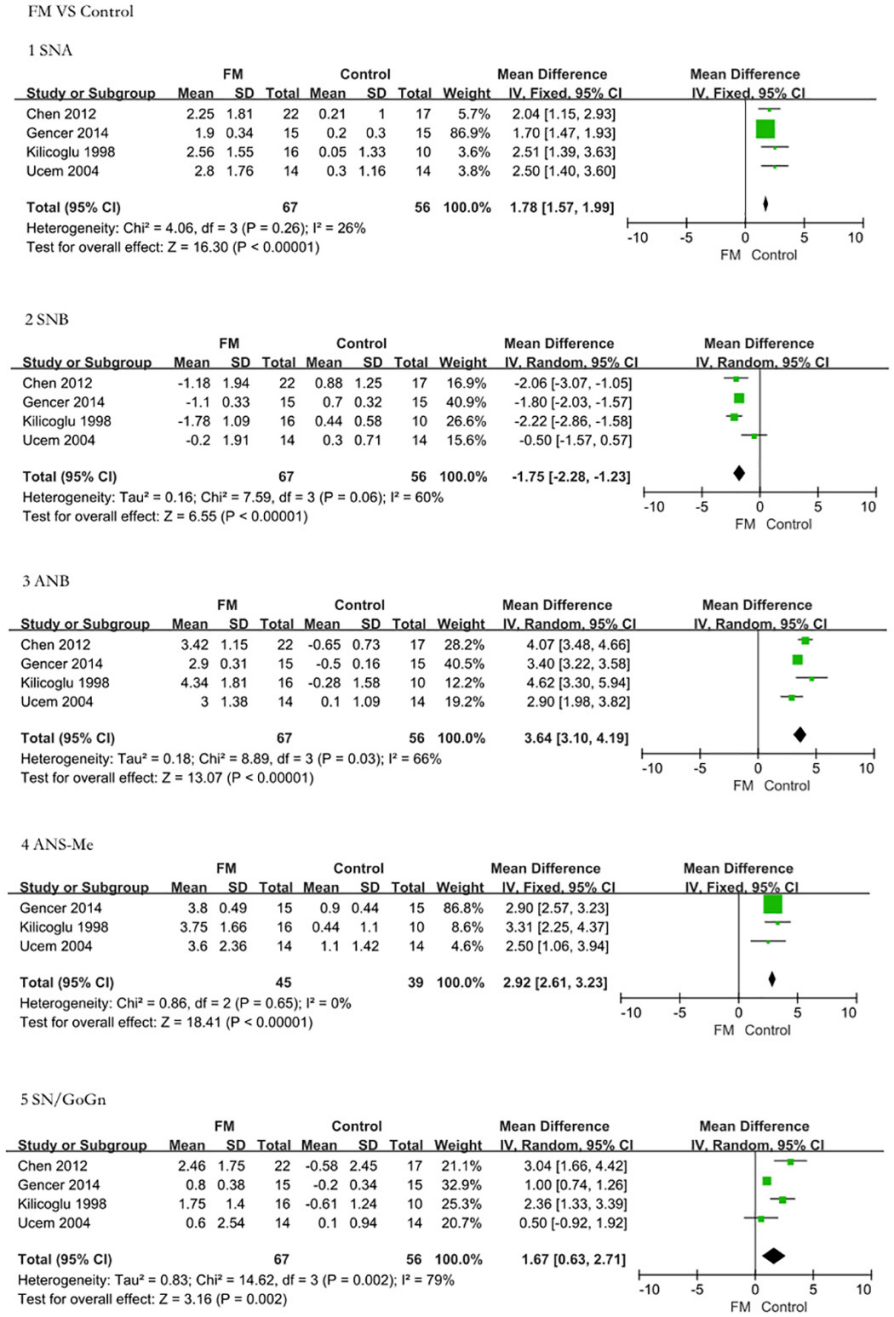
The efficacy assessment of FM treatment for Class III malocclusion versus untreated controls. The comparison was performed using five indices. 1: Angle SNA. The FM treatment group presented a greater increase in SNA than the controls (SMD = 1.78, 95% CI = 1.57–1.99, P < 0.00001). 2: Angle SNB. The FM treatment group presented a greater decrease in SNB than the untreated controls (SMD = -1.75, 95% CI = -2.28–-1.23, P < 0.00001). 3: Angle ANB. The FM treatment group presented a greater increase in ANB than the controls (SMD = 3.64, 95% CI = 3.10–4.19, P < 0.00001). 4: ANS-Me length. The FM treatment group presented a greater increase in ANS-Me length than the controls (SMD = 2.92, 95% CI = 2.61–3.23, P < 0.00001). 5: Angle SN/GoGn. The FM treatment group presented a greater increase in SN/GoGn than the controls (SMD = 1.67, 95% CI = 0.63–2.71, P = 0.002). The FM groups exhibited significant improvement in skeletal retrognathism of the maxilla.

This meta-analysis demonstrated the difference in FM-treated Class III malocclusion patients and controls (Fig 2). We evaluated the five cephalometric parameters most relevant to anterior crossbite compared with those of controls to identify the therapeutic effect and skeletal changes: SNA (SMD = 1.78, 95% CI = 1.57–1.99, P < 0.00001); SNB (SMD = -1.75, 95% CI = -2.28–-1.23, P < 0.00001); ANB (SMD = 3.64, 95% CI = 3.10–4.19, P < 0.00001); SN/GoGn (SMD = 1.67, 95% CI = 0.63–2.71, P = 0.002); and ANS-Me (SMD = 2.92, 95% CI = 2.61–3.23, P < 0.00001). These results suggest that a maxillary protraction appliance can effectively correct anterior crossbite with a retruded maxilla. After FM therapy, the maxilla was displaced anteriorly, whereas the mandible was rotated posteriorly.

### 3 Differences in FM/RME-treated Class III malocclusion patients and untreated controls

**Fig 3:**
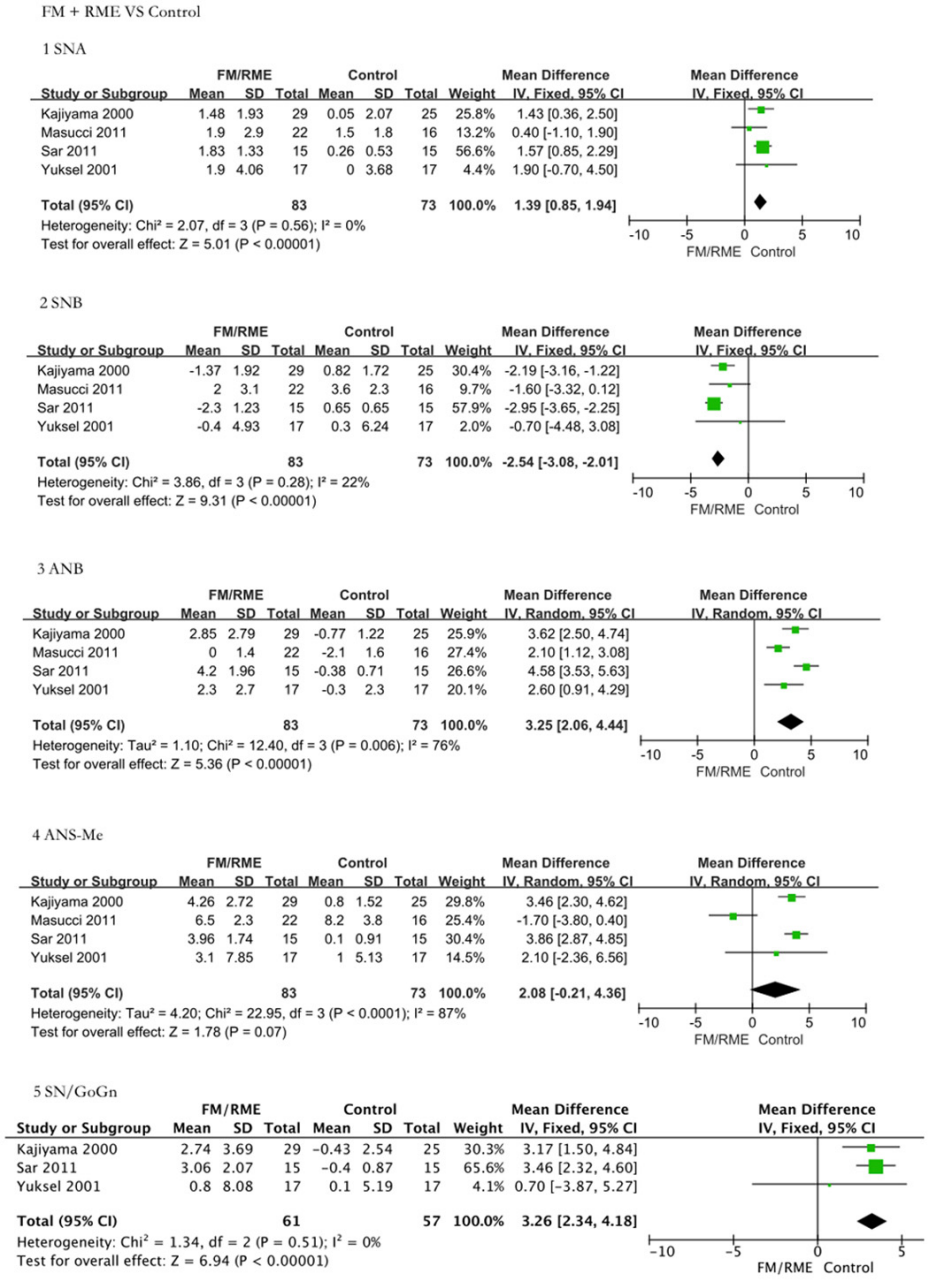
The efficacy assessment of FM/RME treatment for Class III malocclusion versus untreated controls. The comparison was performed using five indices. 1: Angle SNA. The FM treatment group presented a greater increase in SNA than the controls (SMD = 1.39, 95% CI = 0.85–1.94, P < 0.00001). 2: Angle SNB. The FM treatment group presented a greater decrease in SNB than the untreated controls (SMD = -2.54, 95% CI = -3.08–-2.01, P < 0.00001). 3: Angle ANB. The FM treatment group presented a greater increase in ANB than the controls (SMD = 3.25, 95% CI = 2.06–4.44, P < 0.00001). 4: ANS-Me length. The FM treatment group presented a greater increase in ANS-Me length than the controls (SMD = 2.08, 95% CI = -0.21–4.36, P = 0.07). 5: Angle SN/GoGn. The FM treatment group presented a greater increase in SN/GoGn than the controls (SMD = 3.26, 95% CI = 2.34–4.18, P < 0.00001). The FM/RME groups exhibited significant improvement in skeletal retrognathism of the maxilla.

FM/RME-treated Class III malocclusion patients and untreated controls were also compared according to the five most revealing parameters. There was a significant improvement of the sagittal skeletal index in the FM/RME groups (Fig 3). Meta-analysis revealed that FM/RME is an obviously effective method for treating anterior cross-bite patients based on the following results: SNA (SMD = 1.39, 95% CI = 0.85–1.94, P < 0.00001); SNB (SMD = -2.54, 95% CI = -3.08–-2.01, P < 0.00001); ANB (SMD = 3.25, 95% CI = 2.06–4.44, P < 0.00001); SN/GoGn (SMD = 3.26, 95% CI = 2.34–4.18, P < 0.00001); and ANS-Me (SMD = 2.08, 95% CI = -0.21–4.36, P = 0.07).

### 4 Differences between the early treatment group and late treatment group

**Fig 4:**
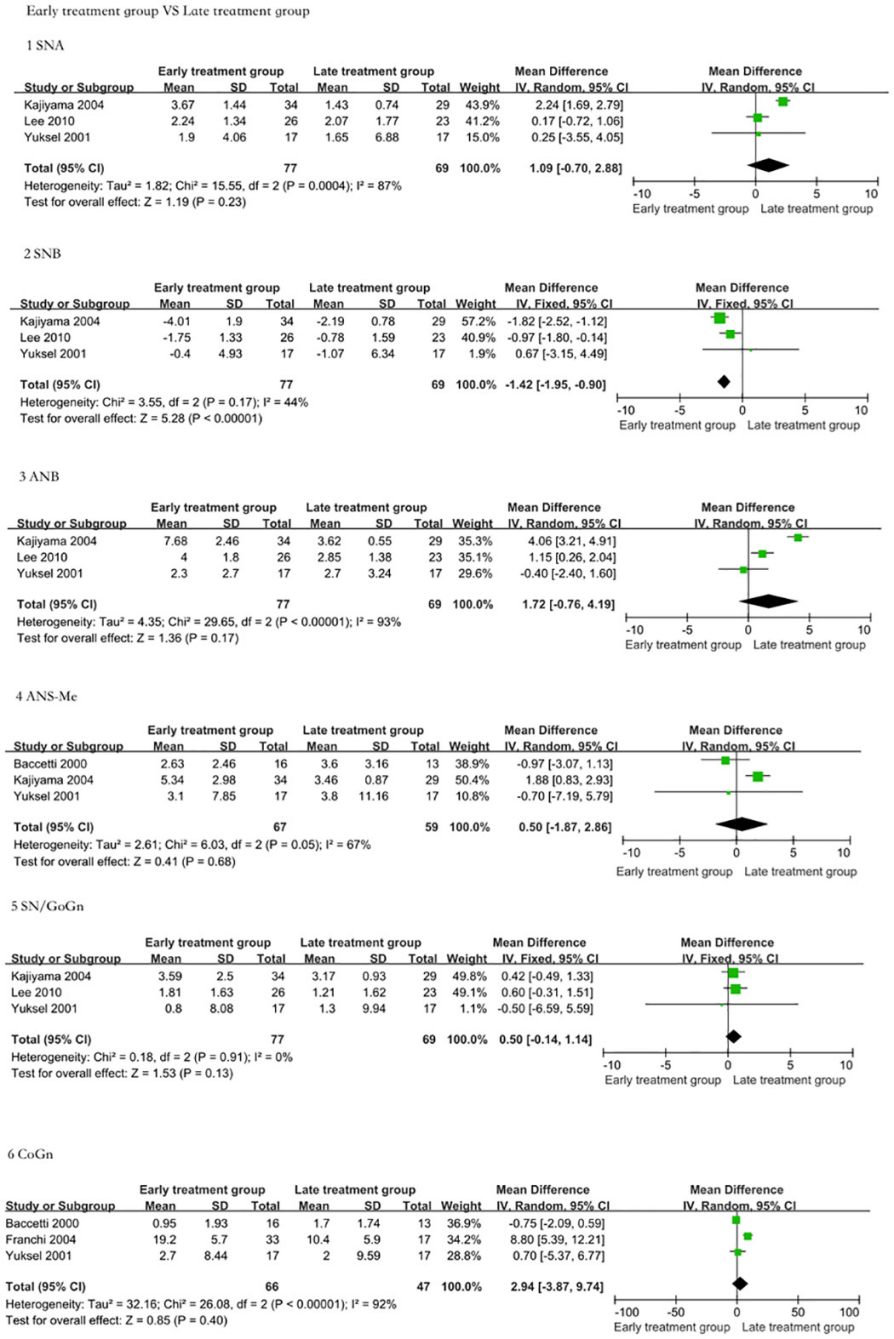
The efficacy assessment of early treatment for Class III malocclusion versus late treatment. The comparison was performed using five indices. 1: Angle SNA. The FM treatment group presented a greater increase in SNA than the controls (SMD = 1.09, 95% CI = -0.70–2.88, P = 0.23). 2: Angle SNB. The FM treatment group presented a greater decrease in SNB than the untreated controls (SMD = -1.42, 95% CI = -1.95–-0.90, P < 0.00001). 3: Angle ANB. The FM treatment group presented a greater increase in ANB than the controls (SMD = 1.72, 95% CI = -0.76–4.19, P = 0.17). 4: ANS-Me length. There were no significant differences in ANS-Me length between the early treatment group and the late treatment group (SMD = 0.50, 95% CI = -1.87–2.86, P = 0.68). 5: SN/GoGn angle. There were no significant differences in SN/GoGn angle between the early treatment group and the late treatment group (SMD = 0.5, 95% CI = -0.14–1.14, P = 0.13). 6: Co-Gn length. There were no significant differences in Co-Gn length between the early treatment group and the late treatment group (SMD = 2.94, 95% CI = -3.78–9.74, P = 0.4). The maxillary protraction effect of the two treatment groups was similar.

A comparison of the early treatment and late treatment groups is shown in Fig 4. Meta-analysis attempted to reveal whether FM therapy yields a greater response to maxillary protraction with an earlier initiation of treatment. Differences were observed in SNA (SMD = 1.09, 95% CI = -0.70–2.88, P = 0.23); SNB (SMD = -1.42, 95% CI = -1.95–-0.90, P < 0.00001); ANB (SMD = 1.72, 95% CI = -0.76–4.19, P = 0.17); SN/GoGn (SMD = 0.5, 95% CI = -0.14–1.14, P = 0.13); Co-Gn (SMD = 2.94, 95% CI = -3.78–9.74, P = 0.4); and ANS-Me (SMD = 0.50, 95% CI = -1.87–2.86, P = 0.68). Following quantitative calculation on the lateral cephalograms, no significant differences were noted between the two groups. In other words, early treatment did not significantly improve modifications in both maxillary and mandibular structures over the results achieved by late treatment group. However, it is even more important to note that some children with permanent teeth can be treated as the late group to improve skeletal crossbite deformity, allowing the avoidance of some unnecessary orthognathic surgeries.

### 5 Sensitivity analysis

**Fig 5:**
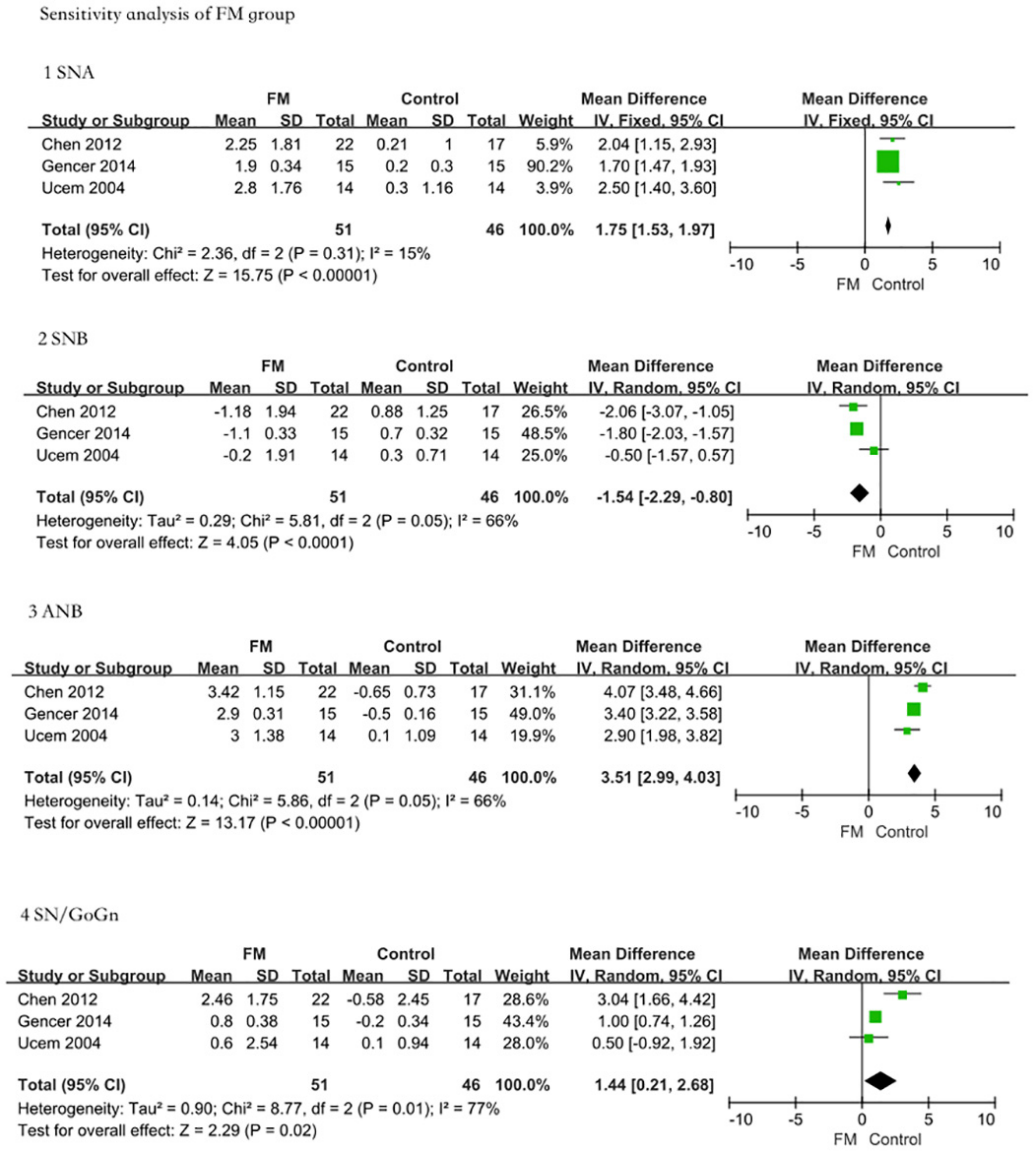
Sensitivity analysis. In the FM group, we chose 3 relatively high-quality studies (score ≥4) to carry out the sensitivity analysis. 1: Angle SNA. The FM treatment group presented a greater increase in SNA than the controls (MD = 1.75, 95% CI = 1.53–1.97, P < 0.00001). 2: Angle SNB. The FM treatment group presented a greater decrease in SNB than the untreated controls (MD = -1.54, 95% CI = -2.29–-0.80, P < 0.0001). 3: Angle ANB. The FM treatment group presented a greater increase in ANB than the controls (MD = 3.51, 95% CI = 2.99–4.03, P < 0.00001). 4: Angle SN/GoGn. The FM treatment group presented a greater increase in SN/GoGn than the controls (MD = 1.44, 95% CI = 0.21–2.68, P = 0.02). The sensitivity analysis results were consistent with previous results.

**Fig 6:**
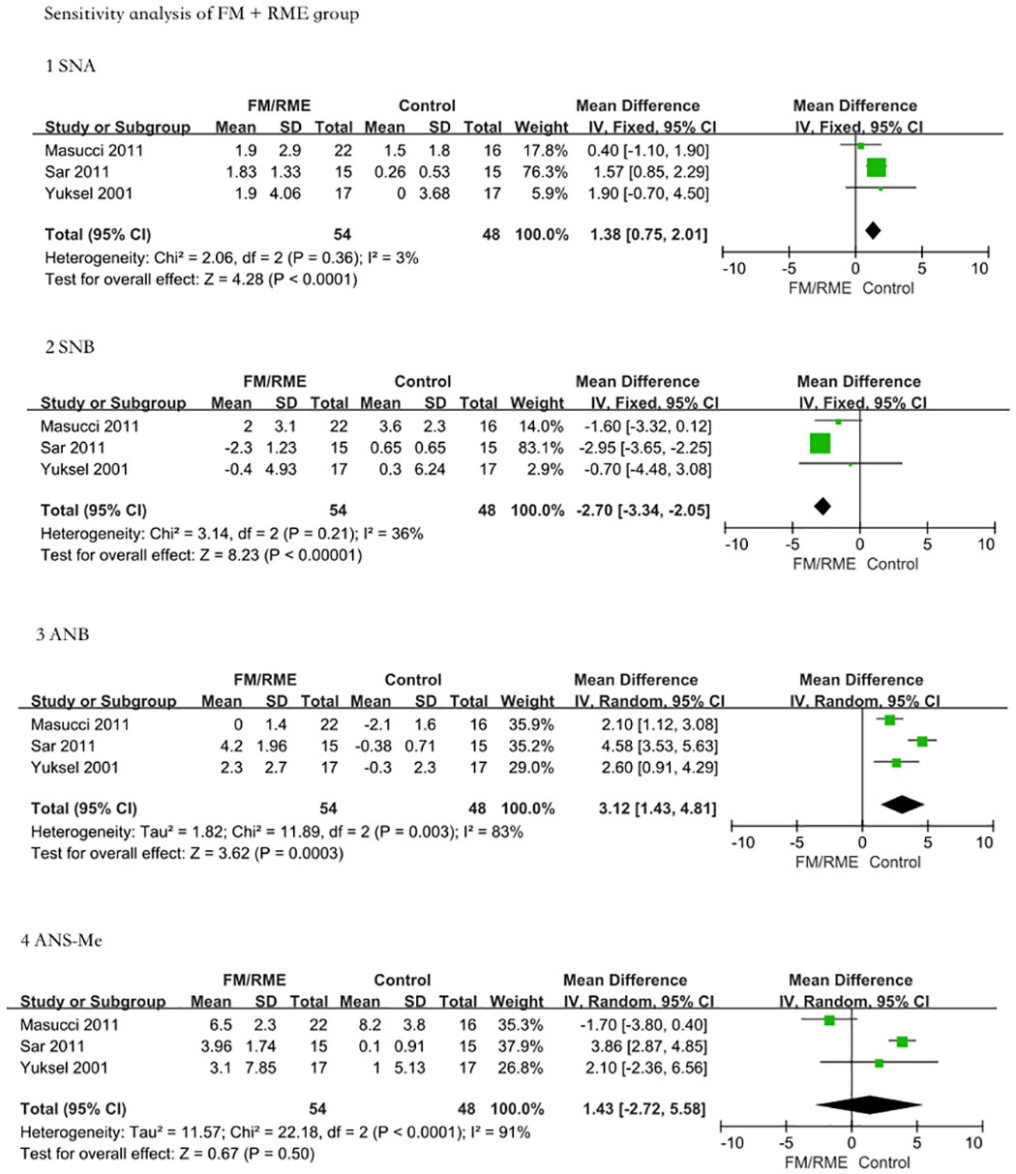
Sensitivity analysis. The same method was used in the FM/RME group; we also chose 3 relatively high-quality studies (score ≥ 4) to carry out the sensitivity analysis. The comparison was performed using five indices. 1: Angle SNA: The FM/RME treatment group presented a greater increase in SNA than the controls (MD = 1.38, 95% CI = 0.75–2.01, P < 0.0001). 2: Angle SNB. The FM/RME treatment group presented a greater decrease in SNB than the untreated controls (MD = -2.70, 95% CI = -3.34–-2.05, P < 0.00001). 3: Angle ANB. The FM/RME treatment group presented a greater increase in ANB than the controls (MD = 3.12, 95% CI = 1.43–4.81, P = 0.0003). 4: ANS-Me length. There were no significant differences in ANS-Me length between the FM/RME treatment group and untreated controls (MD = 1.43, 95% CI = -2.72–5.58, P = 0.50). The sensitivity analysis result was consistent with previous results.

For each measurement index of FM and FM/RME, we chose 3 relatively high-quality studies (score ≥4) to carry out the sensitivity analysis (shown in Figs 5 and 6). Compared with the control group and with the FM group, the maxillary protractor FM groups showed significant changes in the SNA (MD = 1.75, 95% CI = 1.53–1.97, P < 0.00001); SNB (MD = -1.54, 95% CI = -2.29–-0.80, P < 0.0001); ANB (MD = 3.51, 95% CI = 2.99–4.03, P < 0.00001); and SN/GoGn (MD = 1.44, 95% CI = 0.21–2.68, P = 0.02). In addition, the FM/RME treatment group still exhibited effective improvements compared with the control group: SNA (MD = 1.38, 95% CI = 0.75–2.01, P < 0.0001); SNB (MD = -2.70, 95% CI = -3.34–-2.05, P < 0.00001); ANB (MD = 3.12, 95% CI = 1.43–4.81, P = 0.0003); and ANS-Me (MD = 1.43, 95% CI = -2.72–5.58, P = 0.50). The conclusions from the sensitivity analysis were the same as from the previous results, suggesting that FM and FM/RME may be effective early treatments for maxillary dysplasia Class III malocclusion.

### 6 Publication bias

**Fig 7:**
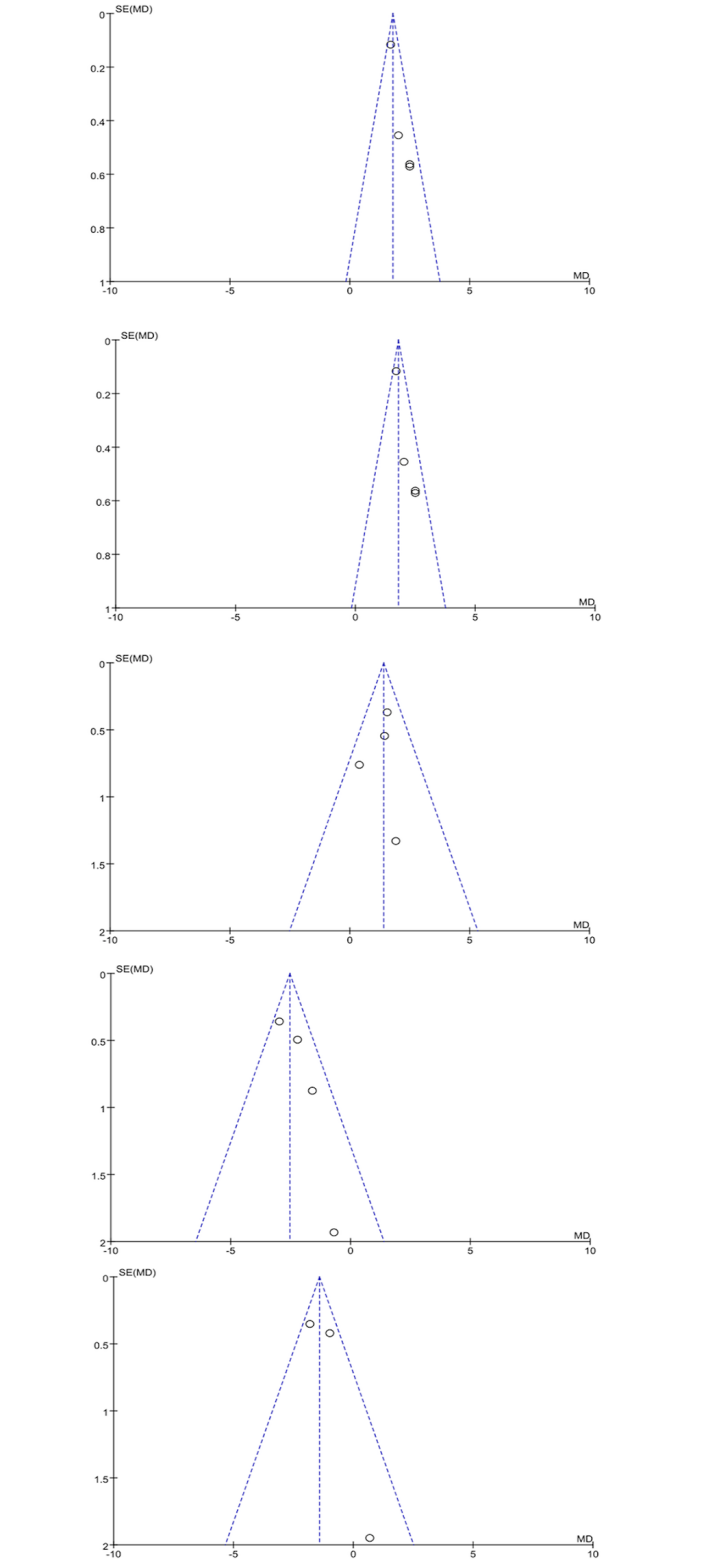
Graphical funnel plots of the included studies. These symmetrical plots indicate the absence of publication bias in the present meta-analysis.

A funnel plot was used to assess the publication bias of the literature. Symmetrical graphical funnel plots were obtained in all included studies (Fig 7).

## Discussion

Treating Class III malocclusion is currently considered one of the most challenging and complex parts of orthodontic practice. According to some surveys, the prevalence of Class III malocclusion is as high as 14% in Asian populations and approximately 1% to 5% in white populations[38–41]. The main causes of skeletal Class III malocclusion are mandibular prognathism or macrognathia, maxillary retrognathism or micrognathia, or a combination of these features. It should be noted that it is far more common to encounter a retruded maxilla than a protruded mandible in skeletal Class III malocclusion[42]. In these cases, orthodontic treatment is needed to protract the maxilla and promote its growth instead of simply limiting mandibular growth to correct the skeletal discrepancy. As a result, the FM is widely used as a feasible and effective maxillary protractor via anterior displacement of the maxilla and redirection of the mandibular position. A review of the literature offers theoretical support for clinical applications, and shows that using the maxillary protraction appliance results in a favorable change in the skeletal relationship when correcting Class III malocclusions[43–48]. From another perspective, several animal studies have shown histological changes of the circumaxillary sutures during anterior displacement[49–52].

If we want to gather adequate evidence to guide clinical practice and establish a standard of treatment, one approach is to treat patients with similar symptoms with the same treatment protocols. However, we cannot deny that definitive conclusions from any one trial are limited and should be treated casually, especially when they are based on studies with limited sample sizes[53,54]. Conversely, meta-analysis provides a reliable technique that can equate and compare research data from several independent, diverse research sources on a specific topic[55–58]. Therefore, a meta-analysis of the relevant literature was performed to determine whether there was support for a consensus viewpoint concerning the efficacy of FM therapy with or without rapid maxillary expansion, as well as the influence of age, treating Class III malocclusion. For the present meta-analysis and after strict elimination of the substandard studies, we only selected 12 independent studies that included a treatment group and naturally growing, untreated Class III controls. To reflect the real effects of FM, the controls are all untreated Class III malocclusion patients rather than individuals without dental malocclusion. There is always disagreement regarding the optimal timing for orthopedic treatment. Many studies have supported early treatment to maximize maxillary anterior advancement, holding the opinion that early FM and expansion therapy is more efficient with early treatment time[37, 59–62]. However, other investigators have held the opposite opinion that there is no relationship between the effect of maxillary FM protraction and treatment timing during pubertal growth[59, 63–66]. This meta-analysis aims to determine if early FM protraction offers any benefit to Class III malocclusion patients over late treatment. It is important that this result be used as evidence to guide clinical practice and to avoid missing cases that can be compensated.

Overall, our meta-analysis aimed to explain three things: firstly, whether the maxilla FM protractor is an effective treatment for skeletal Class III malocclusion; secondly, whether FM protractor and maxilla expansion causes forward displacement of the maxilla and the inhibition of mandibular growth; and thirdly, whether an early treatment group benefits more from maxilla protractor devices than does a parallel late treatment group. The results are as follows: for the first issue, a summary of the meta-analysis suggests that a maxillary protraction appliance is effective for correcting anterior crossbite with a retruded maxilla. The changes in SNA and ANB in the FM group with regard to anterior movement of the maxilla indicate similarity with findings reported in the previous literature [67–70]. The anterior forward rotation of the maxilla was significant in the FM group (P < 0.00001), whereas no significant change was observed in the control group. The skeletal point A forward changes revealed that maxillary growth was achieved in the FM treatment group and that the FM protractor effectively facilitated skeletal growth of the maxilla. The negative change in SNB in the FM group indicates similar results. The negative numerical value change in SNB indicates that mandible growth is limited and that the forward change at the B point is controlled. The effects on combination of SN/GoGn and Me-ANS of the mandibular changes by a maxillary protractor reflect the clockwise rotation of the mandible, which has also been reported in several studies [45, 46, 48, 59, 71]. These results suggest that FM therapy causes the maxilla to be displaced anteriorly, whereas the mandible is rotated posteriorly. The increases of angle SNB (P < 0.00001) and the reduced facial height ANS-Me (P < 0.00001) offer the best evidence supporting this point. Although backward rotation of the mandible plane is an undesired effect of conventional FM therapy, it is inevitable, occurring both in the treatment group and in the Class III untreated subjects. Because the chin serves as the anchorage region in the FM protraction device, a clockwise rotation force was applied directly to the mandible, causing it to be displaced downward and backward during treatment and resulting in an increased mandibular plane angle and reduced facial height. Furthermore, the anterior rotation of the maxilla and growth of the mandible may also contribute to this phenomenon, and a longer treatment time may be another reason. However, we concluded that maxillary protraction FM therapy is an effective early clinical treatment method for skeletal Class III malocclusion. Another question that could arise is how to determine the optimal force characteristics regarding magnitude, duration, and direction to achieve the most effective clinical outcome. One systematic review provided the best clinical evidence. After careful classification and analysis, Yepes et al found that, indeed, no scientific evidence could allow for the definition of adequate parameters for force magnitude, direction, or duration of maxillary protraction FM treatment in Class III patients. However, by clinical consensus, to achieve more efficient treatment effects, they suggested that FMs should be used with 300–400 g of force per side for 14–16 hours per day by applying a force 20 mm greater than the maxillary occlusal plane [72]. According to the research of McNamara and Turley, the RME may disrupt the maxillary sutural system and could be combined with the FM to react as a whole unit [73, 74]. Could the FM applied with rapid maxillary expansion still work in correcting Class III patients, or do the two therapies interrupt each other? We have performed the current analysis to address this question. The outcome of FM/RME is similar to that of FM, indicating that FM/RME is also an effective method to correct skeletal Class III malocclusion. However, the P value of ANS-Me was an exception to this conclusion, which may be attributed to the fact that the treatment group and untreated controls are still developing teenagers, and their changes in growth are therefore not stable. Furthermore, the amount of mandibular skeletal growth is not in equilibrium according to the chronological sequence. Consequently, we are expecting more articles related to our meta-analysis to be published, and we are very interested in exploring the final results.

The results of the last group suggest that early treatment was not more effective than was late FM therapy. An early treatment time involves the early mixed dentition period with the chronologic ages ranging from 7 to 10 years old, whereas a late treatment time is usually applied during late mixed dentition and early permanent teeth dentition from 11 to 14 years old. Evaluation of lateral cephalograms revealed no significant difference between the early treatment group and the late treatment group. In another words, the assumption that orthopedic forces on the maxilla and the mandible were more effective and advantageous during early treatment compared with later treatment was not supported (P > 0.05). The statistical findings offer the best evidence to address the third question. However, with the exception of the p value of SNB (P < 0.05), there is not enough evidence to represent the various angular changes in SNB between the two groups. Considering that the FM therapy patients are all either prepubertal or in puberty, it is expected that the natural growth of the mandible will result in the forward movement of point B and a natural change in SNB. In addition, younger children are expected to have more growth potential than do older children and a more significant change in SNB. As a result, our meta-analysis has provided the best evidence that early treatment is not more effective than late FM therapy. Although there were no differences in treatment time or clinical treatment effects between the early and late groups, the two groups were not entirely the same. In the early treatment patients, the maxillary sutures were still not fully fused at the chronologic ages of 7 to 10 years old. Therefore, the maxillary expansion and protraction effects still included some true skeletal decompensation with new bone deposition at the maxillary sutures. In other cases, the maxillary sutures completely closed after ten years of age. After closure of the maxillary sutures, the expansion of the maxilla lay in the eruption of the maxillary molar teeth and new bone deposition in the buccal side. Therefore, in the late treatment group, dental compensation played the leading role. This finding reminded us that true skeletal decompensation and maxilla expansion are considered possible only in younger children before maxillary suture closure. Although we found that that early treatment was not more effective than late FM therapy, we must admit that orthodontic interference only improves the clinical symptom with dental compensation rather than offering true skeletal decompensation after suture closure of the maxilla. True skeletal decompensation, which is considered possible in younger children, allows for good clinical stability, whereas dental compensation in older children is prone to relapse following appliance removal.

Nonetheless, this study has certain shortcomings similar to other articles due to the nature of meta-analysis. First, the numbers of relevant research articles and patients were not sufficiently large. In addition, some of the relevant studies were excluded from our analysis because of incomplete or overlapping data; consequently, our analysis may not provide a sufficient number of patients/cases. Moreover, not all sources of heterogeneity in the included studies could be addressed. Furthermore, certain methodological limitations exist because a meta-analysis is a retrospective study. Ultimately, we only provide evidence for the effectiveness of FM and FM/RME treatment using untreated controls. What we are also interested in is the difference between the FM- and FM/RME-treated groups. However, the newest relevant and eligible studies of FM groups and FM/RME controls are still not sufficient for conducting a meta-analysis. Given these results, additional research in this field is necessary, and our meta-analyses will continually improve.

## Conclusion

In conclusion, the data of our meta-analysis supported that FM and FM/RME treatment are both effective clinical early treatment methods for skeletal Class III malocclusion. The statistical analysis thoroughly proved that late FM therapy could achieve relatively similar outcomes to early treatment therapy. Although real skeletal development is much better than dental compensation, we cannot abandon maxillary expansion and protraction treatment for early permanent dentition, which we treated as the late group. Current evidence of correlative research still needs to be greatly expanded due to the limited number of published articles in this field. Therefore, we still wish to conduct a large, detailed, randomized, well-designed, comprehensive, controlled trial with a long follow-up visit to confirm our recent research.

## Supporting Information

S1 File The list of full-text excluded articles with the reasons for exclusion. (DOC)

S2 File Checklist. (DOC)
