# Supplementary material for: The Effects of Maxillary Protraction with or without Rapid Maxillary Expansion and Age Factors in Treating Class III Malocclusion: A Meta-Analysis
Source: PLoS One. 2015 Jun 11;10(6):e0130096. doi: 10.1371/journal.pone.0130096 (PMC4466237; doi:10.1371/journal.pone.0130096)
Supplement: S1 File — (DOC) [file pone.0130096.s001.doc]

**The list of full-text excluded articles with the reasons for exclusion.**

**1. A comparison of chincap and maxillary protraction appliances in the treatment of skeletal class III malocclusions.**

R: The treatment method consisted of chincap and maxillary protraction appliances, which is different from our facemask therapy with or without a rapid maxillary protractor.

# 2. A maxillary protracting bow appliance for Class III treatment in the primary dentition.

# R: The study illustrated only two cases. It is a case report.

# 3. A comparison of two different techniques for early correction of Class III malocclusion.

R: The subjects consisted of cases undergoing either Reverse Twin-Block (RTB) therapy or protraction face mask (PFM) treatment. We enrolled PFM therapy patients only.

# 4. A new approach to correct a Class III malocclusion with miniscrews: a case report.

# R: This is a case report.

# 5. Bimaxillary surgery in Class III malocclusion: Soft and hard tissue changes.

# R: In this study, Class III malocclusion patients underwent Le Fort I maxillary advancement and mandibular setback surgery with sagittal split osteotomy, rather than orthodontic treatment.

# 6. Bone- and dentoalveolar-anchored dentofacial orthopedics for Class III malocclusion: New approaches, similar objectives?

# R: The method in this study differed from ours.

# 7. Case report: skeletal Class III correction by orthodontic means.

# R: This is a case report.

# 8. Combined rapid maxillary expansion and protraction facemask in the treatment of class III malocclusions in growing children: A prospective long-term study.

R: This is a prospective study.

**9. Clinical results of the maxillary protraction in Korean children.**

R: First, our article was not intended to discuss the treatment effects of FM in different races. In addition, the comparison in this study was between an RPE treatment group and a La/Li treatment group; in contrast, wanted to discuss the differences in treatment groups and untreated controls.

# 10. Clinical study of maxillary protraction combined with maxillary molar distalization appliance therapy on cross bite and crowding malocclusion

R: This study treated malocclusion patients with maxillary protraction and maxillary molar distalization appliances. We only enrolled maxillary protraction-treated patients.

# 11. Correction of skeletal Class III malocclusion with clockwise rotation of the maxillomandibular complex.

# R: This is a case report.

# 12. Craniofacial morphology of skeletal class III patients before treatment and growth-related changes during treatment with a maxillary protraction appliance: a comparison of orthodontic and surgical cases.

# R: The comparison in this study was between orthodontic and surgical groups. We compared an orthodontic treatment group to untreated controls.

# 13. Cephalometric A point changes during and after maxillary protraction and expansion.

# R: This study only analyzed cephalometric A point changes; therefore, the amount of data was not sufficient.

# 14. Dentoskeletal effects and facial profile changes in Class III patient treated with protraction facemask appliance: a case report.

# R: This is a case report.

# 15. Dentofacial effects of two facemask therapies for maxillary protraction Miniscrew implants versus rapid maxillary expanders.

# R: This study compared the treatment method of facemask in association with miniscrew implants (MSI/FM) and a facemask with rapid maxillary expanders (RME/FM), whereas we wanted to compare FM or FM/RME with untreated controls.

# 16. Dentofacial orthopaedic correction of maxillary retrusion with the protraction facemask--a literature review.

# R: This is a literature review.

# 17. Exploitation of the residual premaxillary-maxillary suture site in maxillary protraction. An hypothesis.

# R: This is a discussion that originated from a case report.

**18. Early Treatment Protocol for Skeletal Class III Malocclusion**

R: This study included only one case.

**19. Early treatment of Class III malocclusion: Is it worth the burden?**

R: This analytical article was not a case-control study or a randomized, controlled trial (RCT).

**20. Effects of maxillary protraction for early correction of class III malocclusion.**

R: This article discussed Class III subjects with maxillary retrusion and mandibular protrusion and compared these changes with those of an untreated, well-matched control sample with normal occlusions. In contrast, we enrolled untreated Class III malocclusion patients as controls.

# 21. Effects induced after the use of maxillary protraction appliances: a literature review.

R: This is a review.

**22. Expansion/Facemask Treatment of an Adult Class III Malocclusion.**

R: This is a case report.

**23. Efficacy of orthopedic treatment with protraction facemask on skeletal Class III malocclusion a systematic review and meta-analysis.**

R: This systematic review was not a case-control study or an RCT. The study did not provide original data.

**24. Evaluation of treatment and posttreatment changes of protraction facemask treatment using the PAR index.**

R: This study used the Peer Assessment Rating (PAR) index score to evaluate the treatment and posttreatment changes in Class III patients treated by protraction facemask. However, we used the five cephalometric parameters most relevant to anterior crossbite for our evaluation.

**25. Face mask protraction therapy in early skeletal class III malocclusion.**

R: This study did not provided original data.

**26. Geometric morphometric assessment of treatment effects of maxillary protraction combined with chin cup appliance on the maxillofacial complex.**

R: In this study, the treatment method was maxillary protraction combined with chin cup, which differed from our selected standards.

# 27. Influence of intentional ankylosis of deciduous canines to reinforce the anchorage for maxillary protraction.

R: The treatment method was not FM or FM/RME.

# 28. Growth modification treatment in class III malocclusions--an orthodontic case report.

R: The study is a case report.

**29. Long-term effects of Class III treatment with rapid maxillary expansion and facemask
therapy followed by fixed appliances.**

R: This article discussed the long-term effects and stability of rapid maxillary expansion and facemask (RME/FM); therefore, it was not in conformity with our center.

**30. Long-term stability of Class III treatment: Rapid palatal expansion and protraction
facemask vs LeFort I maxillary advancement osteotomy.**

R: The aim of this retrospective cephalometric study was to compare the long-term stability of early protraction facemask treatment with later surgical maxillary advancement with LeFort I osteotomy, which was not used in our article.

**31. Managing the developing Class III malocclusion with palatal expansion and facemask therapy.**

R: This article was not a case-control study or an RCT. The study did not provide original data.

**32. Morphometric analysis of treatment effects of bone-anchored maxillary protraction in growing Class III patients.**

R: This article used bone anchored maxillary protraction (BAMP) for the treatment of growing patients with Class III malocclusions, which was different from our methods.

# 33. Maxillary protraction on Class III malocclusion: A prospective study.

# R: This was a prospective study.

# 34. Maxillary protraction with miniplates providing skeletal anchorage in a growing Class III patient.

# R: This is a case report.

# 35. MnBillnry protraction treatment of skeletal Class III children using miniplnte anchorage.

# R: The treatment method in this study was miniplate anchorage, and its original language was not English.

# 36. Masticatory muscle pain before, during, and after treatment with orthopedic protraction headgear: a pilot study

# R: The study was a pilot study that did not meet the standards.

# 37. Modified maxillary protraction headgear for the correction of class III skeletal malocclusion with anterior open bite.

# R: The patients had Class III skeletal malocclusions with anterior open bites in this study. In contrast, we only enrolled Class III skeletal malocclusion patients without other cases of malocclusion.

# 38. Maxillary protraction for early orthopedic correction of skeletal ClassIII malocclusion.

# R: The study did not provide original data, and it was not a case-control study or an RCT.

# 39. MnBillnry protraction treatment of skeletal Class III children using miniplnte anchorage

# R: The study is a case report, and its original language was Korean.

# 40. Nonextraction treatment of a skeletal Class III adolescent girl with expansion and facemask: Long-term stability.

# R: This is a case report. In addition, the author wanted to evaluate the combined use of maxillary expansion and a protraction facemask in the correction of a skeletal Class III malocclusion after the patient's pubertal growth spurt, which was different from our aim.

# 41. Non-compliant maxillary protraction by orthodontic micro-implants.

# R: The curative method in this study was a new monomaxillary, fixed, implant-supported, aesthetically pleasing type of micro-implant device.

# 42. Orthopaedic treatment efficiency in skeletal Class III malocclusions in young patients: RME-face mask versus TSME.

# R: This study compared the treatment method of a transverse sagittal maxillary expander (TSME) appliance (Group 1) and a Hyrax rapid maxillary expander/facemask (RME/FM, Group 2); in contrast, we compared FM or FM/RPE with untreated controls.

# 42. Orthodontic decompensation and correction of skeletal Class III malocclusion with gradual dentoalveolar remodeling in a growing patient.

R: This was a case analysis including only an 8-year-old girl with a skeletal Class III malocclusion.

**44. Orthopedic Treatment Outcomes in Class III Malocclusion**

R: This is a systematic review.

**45. Orthopaedic Protraction of the Maxilla with Miniplates: Treatment of Midface Deficiency.**

R: The article included only two cases with class III malocclusion and maxillary deficiency treated using titanium miniplates, which differed from our treatment methods.

# 46. Orthodontic correction of a class III malocclusion in an adolescent patient with a bonded RPE and protraction face mask

R: The article was a case report.

**47. Optimal force for maxillary protraction facemask therapy in the early treatment of class III malocclusion.**

R: This is a systematic review.

**48. Preoperative incisor inclination in patients with Class III dentofacial deformities treated with orthognathic surgery.**

R: The treatment of orthognathic surgery has nothing to do with our topic.

**49. Miniplate implant anchorage for maxillary protraction in Class III malocclusion.**

R: In this article, the author used miniplate implants as anchorage for maxillary protraction in skeletal Class III malocclusions with retruded maxilla. Additionally, the original language of the study was not English.

# 50. Rapid maxillary expansion compared to surgery for assistance in maxillary face mask protraction.

# R: This study compared the effectiveness of two different methods, which were rapid maxillary expansion (RME) plus face mask (FM) and LeFort 1 osteotomy + FM during maxillary protraction; in contrast, we compared a facemask or facemask and rapid maxillary expansion with untreated controls.

**51. Skeletal anchored maxillary protraction for midface deficiency in children and early adolescents with Class III malocclusion: A systematic review and meta-analysis.**

R: The paper is a systematic review.

**52. Skeletal and Dental Effects of a Mini Maxillary Protraction Appliance.**

R: First, the Class III patients were treated with a modified protractor, which was different from our traditional face mask therapy. In addition, untreated children with Class I malocclusions were selected as the control group, whereas we choose untreated Class III patients as untreated controls.

**53. Skeletal and dental effects of maxillary protraction in patients with angle class III malocclusion. A meta-analysis.**

R: The paper is a meta-analysis.

**54. Surgically assisted rapid maxillary expansion (SARME): a review of the literature.**

R: The paper is a review.

**55. Soft tissue profile changes following maxillary protraction in Class III subjects.**

R: This paper discussed soft tissue profile changes. In contrast, we examined skeletal index changes.

**56. The effectiveness of protraction face mask therapy: A meta-analysis.**

R: This is a meta-analysis.

**57. The effects of face mask and tongue plate on maxillary deficiency in growing patients: a randomized clinical trial.**

R: This paper is an RCT. However, the experimental group was treated with a removable facemask and the control group with a tongue plate. The contrast of the two groups was not related to our topic.

# 58. The effect of two different methods of rapid maxillary expansion on treatment results of skeletal Class III malocclusion patients with maxillary protraction in early permanent dentition.

# R: The author sought to evaluate the differences between single rapid maxillary expansion and repetitive rapid expansion and constriction with maxillary protraction in treating early permanent skeletal Class III patients. Therefore, the comparison involved two different treatment methods.

# 59. The clinical study of protraction face mask combining with MEAW technique to correct skeletal Class III malocclusion in the early permanent tooth.

# R: The method in this paper was the combination of multiloop edgewise arch wire (MEAW) with a protraction facemask, which differed from our methods.

# 60. The short-term treatment effects of face mask therapy in Class III patients based on the anchorage device: miniplates vs rapid maxillary expansion.

# R: The treatment methods in this study were face mask therapy with miniplates (FM-MP) and face mask therapy with rapid maxillary expansion appliance (FM-RME).

# 61. The treatment of Class III: systematic review - Part I. Magnitude, direction and duration of the forces in the maxillary protraction.

# R: This is a systematic review.

# 62. Treatment of Class III malocclusion in a young adult patient: a case report.

R: This is a case report.

# 63. Treatment of a Class III malocclusion as an example of Camouflage-Therapy. A case report.

R: This is a case report.

# 64. Three-dimensional analysis of maxillary changes associated with facemask and rapid maxillary expansion compared with bone anchored maxillary protraction.

# R: The rapid maxillary expansion (RME/FM) group was compared with bone-anchored maxillary protraction (BAMP), rather than untreated controls.

# 65. Three-dimensional assessment of mandibular and glenoid fossa changes after bone-anchored Class III intermaxillary traction.

# R: The treatment method in this study was bone-anchored maxillary protraction, whereas we used facemask extraoral devices.

# 66. Three-dimensional assessment of maxillary changes associated with bone anchored maxillary protraction

R: This study evaluated 3-dimensional changes in the maxilla, the surrounding hard and soft tissues, and circummaxillary sutures after bone-anchored maxillary protraction treatment. It therefore differed from our purposes.

# 67. The maxillary protraction treatment: description of a laser Er:Yag-assisted surgical technique. Case report.

R: The study is a case report.

# 68. Treatment and posttreatment effects of a facial mask combined with a bite-block appliance in Class III malocclusion.

# R: The treatment group used a facial mask combined with a removable bite-block appliance.

# 69. Treatment of a young adult with Class III malocclusion using a modified mini maxillary protractor: a case report

R: This is a case report.

# 70. Use of intermaxillary forces in early treatment of maxillary deficient Class III patients: Results of a case series

R: The patients were treated with modified lower acrylic resin plates bonded on the mandibular tooth surfaces, which are different from traditional facemasks.

**71. Long-term results of surgically assisted maxillary protraction vs regular facemask.**

R: This article compared the short- and long-term treatment results of rapid maxillary expansion (RME) and surgical assistance during maxillary protraction with a facemask (FM). We discussed the differences between treatment groups and untreated controls in RME treatment.

**72. Successful treatment of early class III malocclusion with protraction headgear.**

R: This is a case report.

# 73. Effect of protraction headgear on Class III malocclusion.

# R: This passage only included ten patients with skeletal Class III malocclusion. In addition, the patients were all in early mixed dentition, negating the ability to compare early and late treatment periods. Third, the control group did not consist of untreated Class III malocclusion patients.

# 74. Treatment of class III Malocclusion with maxillary expansion and face-mask therapy: A case report.

R: This is a case report.
